# Concepts for brain aging: resistance, resilience, reserve, and compensation

**DOI:** 10.1186/s13195-019-0479-y

**Published:** 2019-03-11

**Authors:** Thomas J. Montine, Brenna A. Cholerton, Maria M. Corrada, Steven D. Edland, Margaret E. Flanagan, Laura S. Hemmy, Claudia H. Kawas, Lon R. White

**Affiliations:** 1 Department of Pathology, Stanford University, Palo Alto, CA USA; 2 Department of Neurology, UC Irvine, Irvine, CA USA; 3 Department of Family Medicine and Public Health, UC San Diego, La Jolla, USA; 4 Department of Laboratory Medicine and Pathology, University of Minnesota, Minneapolis, MN USA; 5 Pacific Health Research and Education Institute, Honolulu, HI USA; 6 GRECC, Minneapolis VA Health Care System, University of Minnesota, Minneapolis, MN USA; 7 Department of Neurosciences, UC San Diego, La Jolla, USA

**Keywords:** Reserve capacity, Resistance, Resilience, Alzheimer’s disease

## Abstract

A primary goal of research in cognitive impairment and dementia is to understand how some individuals retain sufficient cognitive function for a fulfilling life while many others are robbed of their independence, sometimes their essence, in the last years and decades of life. In this commentary, we propose operational definitions of the types of factors that may help individuals retain cognitive function with aging. We propose operational definitions of *resistance*, *resilience*, *reserve*, with an eye toward how these may be measured and interpreted, and how they may enable research aimed at prevention. With operational definitions and quantification of resistance, resilience, and reserve, a focused analytic search for their determinants and correlates can be undertaken. This approach, essentially a search to identify protective risk factors and their mechanisms, represents a relatively unexplored pathway toward the identification of candidate preventive interventions.

## Commentary

A primary goal of research in cognitive impairment and dementia is to understand how some individuals retain sufficient cognitive function for a fulfilling life while many others are robbed of their independence, sometimes their essence, in the last years and decades of life. Here, we propose to define key concepts for which there is not yet a consensus. At the outset, we recognize that our focus is biological (molecules, cells, systems, organism), appreciate the major impact of environmental and social determinants of health and admit our prejudice that environmental and social factors ultimately impact cognition through biological processes.

**Fig. 1:**
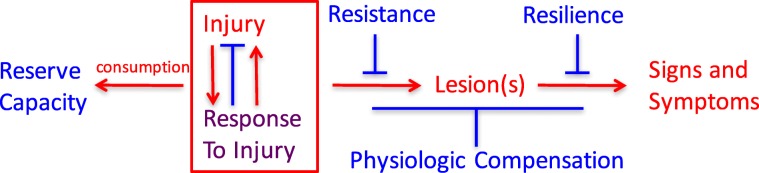
Relationships among adverse (red), protective (blue), and mixed (purple) processes that culminate in signs and symptoms of neurodegenerative diseases

It seems likely that a host of diverse factors active during fetal development, childhood, and throughout adult life may initiate, aggravate, or protect against relevant pathophysiologic processes that underlie neurodegeneration and its clinical expression. These factors—some adverse and some protective—may operate independently, synergistically, antagonistically, sequentially, or even differentially (Fig. 1). While some may be examined individually and in exquisite molecular detail in animal or in vitro models, most will require careful, longitudinal validation in humans. From this perspective, it is not surprising that so far we have had only limited success in identifying risk factors and their underlying mechanisms to guide effective primary and secondary preventive interventions.

Until quite recently, “late onset Alzheimer’s disease” was widely viewed as a specific disease entity responsible for the vast majority of late-life dementia. However, longitudinal epidemiologic studies of brain aging and cognitive decline with brain autopsy have consistently demonstrated a central role for multiple co-morbidities as the dominant determinants of late-life dementia. It is important to recognize that current intra vitam measures of these several common diseases of the aging brain are limited, and consequently, despite limitations, brain histopathologic evaluation remains the only means to assess comprehensively the impact of co-morbid diseases on cognitive performance during life.

In combination with functional assessments obtained during life, histopathologic features (lesions) determined with brain autopsy define the presence of specific clinico-pathologic entities, which may or may not reliably correspond to specific mechanism(s) of disease. As a result of the highly consistent findings from longitudinal epidemiologic studies with brain autopsy from across the globe, the view of cognitive decline and dementia in older adults is shifting from being the result of a single disease to a conspiracy of multiple, common age-related disease processes that combine idiosyncratically in each individual. The most common is Alzheimer’s disease, defined by amyloid beta accumulation and neurofibrillary degeneration in certain regions of the brain. Four other commonly recognized pathophysiologic processes that can contribute to cognitive decline and dementia in late life include Lewy body disease, vascular brain injury (especially from small vessel disease), hippocampal sclerosis, and generalized atrophy beyond what can be explained by these other diseases. While the brain lesions of AD are more prevalent at autopsy than any of the other lesions, the combined frequencies of the non-AD abnormalities are usually greater. Indeed, in both the Nun Study and the Honolulu Asia Aging Study, > 90% of participants with severe cognitive impairment can be fully attributed to the collective or individual influences of these five abnormalities [1]. It is critically important, but infrequently appreciated, that the exponential influence of co-morbid disease is reflected in the multiplication of individual relative risks (or odds ratios) for each disease related to cognitive impairment or dementia (Table 1).

**Table 1:** Point estimates of odds ratios (OR) from ordinal logistic regression of the impact of the coprevalence of five brain lesions on cognitive performance within 2 years of death

Lesion co-morbidity index	OR for the Nun Study (*n* = 334)	OR for the Honolulu Asia Aging Study (*n* = 774)
0	1.0 (reference)	1.0 (reference)
0.4–0.8	2.8	2.4
1.0–1.8	5.0	4.6
2.0–2.4	23.1	16.3
2.6–4.4	99.1	37.6

Severity of each of the five brain lesions (Braak stage for neurofibrillary degeneration, cerebral cortical Lewy body disease, cerebral cortical microinfarcts, hippocampal sclerosis, low brain weight) was scored as none/mild (0), moderate (0.4), or severe (1.0) by established criteria, and the lesion co-morbidity index was calculated as the sum of scores for each of the five lesions [1]

To frame a discussion of resistance, resilience, reserve, and compensation, we conventionally consider the diseases that cause late-life cognitive impairment and dementia to derive from injury and response to injury that begin before there are signs or symptoms, but that the resulting damage, distortion, disruption, and/or degeneration ultimately becomes overwhelmingly evident as impairments of cognitive and behavioral function.

The recognition of risk factors linked to measures of different types and amount of brain lesions may illuminate fundamental mechanisms and primary instigating exposures. A systematic search to identify specific protective factors and the mechanisms that underlie them has been conducted relatively infrequently. We propose the following operational definitions as a step toward systematically investigating each of these processes in individuals:

*Resistance* is inferred from an observed absence or lower level of dementia-associated brain injury, relative to an expected greater frequency or severity based on age, genetic factors, or other characteristics of the individual. This state of unexpectedly low or absent brain injury theoretically may be *intrinsic*, meaning in someone with greater defenses to forces that usually lead to brain lesions, or *environmental*, meaning in someone with usual defenses but who avoided exposure to these forces. While resistance now can be assessed comprehensively only with neuropathologic evaluation, specific facets (e.g., beta amyloid, pathologic tau burden, neuron damage) can be estimated during life with biomarkers and imaging.

*Resilience* is inferred from an observed level of cognitive functioning higher than expected in the face of demonstrated brain injury. Resilience only can be recognized or measured when injury exists and can be related to (near) coincident assessment of function. We prefer to consider two forms of resilience: apparent and essential. *Apparent resilience* refers to a specific lesion type without consideration of common co-morbidities. Consider two individuals who both are positive by PET imaging for fibrillar amyloid and pathologic tau; one is cognitively normal and the other has dementia. The first person has apparent resilience to AD neuropathologic change. Imagine further a future state when there also is a PET ligand for pathologic alpha-synuclein. Now, we learn that the first person lacks Lewy body disease and the second has co-morbid neocortical Lewy body disease. Is the difference between these two individuals explained by resilience to AD neuropathologic change or by resistance to Lewy body in the first person? Once comprehensive assessment of brain lesions associated with dementia is achieved, then *essential resilience* can be evaluated. Currently, this is accomplished best with neuropathologic assessment, but even this approach is limited. Our brain autopsy data suggest that much, and perhaps most, of what is referred to currently as (apparent) resilience actually is resistance to co-morbid disease.

Consumption or retention of *reserve* can be measured or inferred either as brain structural and/or physiological pre-morbid capacity. Examples might be greater than usual synaptic density (analogous to computational “hardware”) or enhanced cognitive effectiveness or redundancy because of learned language, educational richness, or occupational complexity (analogous to computational “software”) prior to the onset of disease. The salutary influence of such resources may be apparent in cognitive test performance well before the onset of cognitive decline. This definition requires that measures of reserve capacity must have been estimated or inferred prior to the development of brain injury. Mechanisms underlying physiologic *compensation* also may be changes in “hardware” or “software,” but in distinction to pre-existing reserve capacity, physiologic compensation occurs following injury rather than developing prior to injury/response to injury. An example of physiologic compensation might be recruitment of additional regions of the brain to subserve memory function following damage to the hippocampus or in recovery of language functioning after an infarction or brain injury.

## Conclusions

With operational definitions of resistance, resilience, and reserve, a focused analytic search for their predictors and correlates can be undertaken. This will require distinguishing and measuring each independently, and then employing those measures as distinct endpoints to identify their individual determinants. This approach, essentially a search to identify protective risk factors and their mechanisms, represents a relatively unexplored pathway toward the identification of candidate preventive interventions.
